# Nocturnal enuresis associated with lithium carbonate extended-release tablets in a girl with major depressive disorder: A case report

**DOI:** 10.1097/MD.0000000000042505

**Published:** 2025-05-16

**Authors:** Yanping Feng, Qingqing Xiang, Xianzhen Dai, Pingping Ma, Bo Liu, Youguo Tan

**Affiliations:** a The Zigong Affiliated Hospital, Southwest Medical University, Zigong, China; b Department of Auxiliary Examination, Zigong Mental Health Center, Zigong, China; c Child and Adolescent Psychosomatic Medicine Center, Zigong Mental Health Center, Zigong, China; d Research Center for Psychiatry, Zigong Institute of Brain Science, Zigong, China.

**Keywords:** adolescent, lithium carbonate extended-release tablet, major depressive disorder, nocturnal enuresis, psychotropics

## Abstract

**Rationale:**

Lithium carbonate, a mood stabilizer, is most commonly used for the treatment and prevention of mania and bipolar disorder. Common renal side effects include polyuria and polydipsia. However, when combined with other psychotropic medications that have sedative properties, lithium may increase the risk of nocturnal enuresis.

**Patient concerns:**

A 12-year-old girl experienced recurrent major depressive episodes with psychotic symptoms. Her clinical presentation was complex, including depressed mood, irritability, suicidal ideation or behavior, and partial psychotic features. During treatment, she frequently reported polydipsia, polyuria, and nocturnal enuresis.

**Diagnoses:**

Based on the International Classification of Diseases, 10th Edition, the patient was diagnosed with major depressive disorder with psychotic symptoms (F32.3) and enuresis not due to a physical disorder (F98.0).

**Interventions:**

In addition to receiving antidepressants, antipsychotics, benzodiazepines, and modified electroconvulsive therapy, she was also intermittently treated with lithium carbonate extended-release tablets, with a maximum daily dose of 1.2 g. No pathophysiological abnormalities were identified through imaging examinations in relation to her nocturnal enuresis. After suspecting a link between her enuresis and lithium, her nocturnal enuresis resolved following the discontinuation of lithium carbonate extended-release tablets.

**Outcomes:**

During the 4-month follow-up, the patient remained free of nocturnal enuresis, while her depressive and psychotic symptoms significantly subsided.

**Lessons:**

Psychiatrists should be aware of the possibility of lithium-induced nocturnal enuresis when polyuria and polydipsia emerge during lithium treatment, particularly in children and adolescents receiving concomitant psychotropic medications with sedative properties.

## 1. Introduction

Nocturnal enuresis is characterized by intermittent urinary incontinence during sleep in children aged ≥ 5 years, occurring at least once a month for at least 3 months.^[1]^ In the mental health field, medication-related enuresis has been linked to various types of psychotropics, including antidepressants,^[2,3]^ antipsychotics,^[4,5]^ antiepileptics,^[6,7]^ and benzodiazepines.^[8]^ For instance, a 55-year-old patient receiving mirtazapine treatment developed enuresis.^[9]^ A small-scale clinical study of 61 Chinese in-patients reported that the incidence of clozapine-induced enuresis was as high as approximately 44%.^[10]^ Additionally, a prospective study of 72 children found a secondary enuresis rate of approximately 24% after a mean of 19.8 days of valproate exposure.^[6]^

Lithium carbonate, as a mood stabilizer, is approved by the Food and Drug Administration as the first-line treatment for bipolar disorder.^[11]^ It is also effective in reducing the recurrence of depressive episodes and in preventing suicide.^[12]^ Lithium toxicity can occur at doses close to therapeutic levels. The therapeutic range for blood lithium concentration is between 0.6 and 1.2 mmol/L,^[13]^ with toxicity typically observed at levels above 1.4 mmol/L.^[14]^ The most common toxic effects include gastrointestinal symptoms, such as nausea, vomiting, and diarrhea; neurological symptoms, such as tremors, ataxia, and delirium; and renal symptoms, such as polyuria and polydipsia.^[15]^ Lithium-related enuresis is, however, relatively uncommon compared to other psychotropics. A previous randomized controlled trial involving 60 pediatric patients with bipolar disorder reported that the incidence of lithium-related enuresis was as high as 30%.^[16]^ Another early randomized controlled trial involving 91 children with conduct disorder reported that the incidence of lithium-related enuresis was 17.4%.^[17]^ Due to improved monitoring of blood lithium concentrations, reports of lithium-related enuresis have become rare in recent years. Furthermore, no reports to date have specifically addressed cases of nocturnal enuresis associated with lithium carbonate extended-release tablets.

Here, we report a rare case of nocturnal enuresis associated with lithium carbonate extended-release tablets in a girl diagnosed with major depressive disorder. In addition to lithium, she was also treated with antidepressants, antipsychotics, and benzodiazepines. We propose that her nocturnal enuresis may have resulted from an interaction effect between lithium-induced polyuria and the sedative properties of concomitant psychotropic agents.

## 2. Case presentation

**Figure 1.**
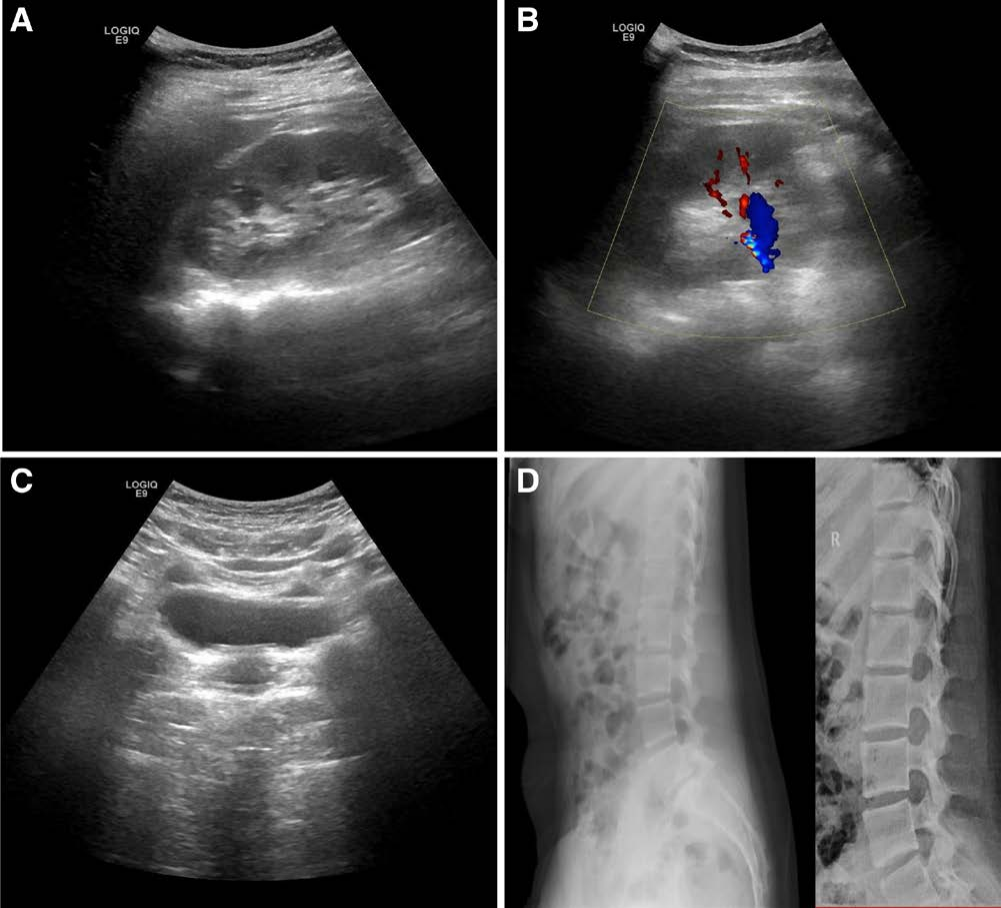
Urological system ultrasonic image and spine bone digital X-ray imaging. No abnormalities were detected. (A) Image of renal structure; (B) image of renal blood stream; (C) bladder image; (D) image of spine bone.

On November 10, 2023, a 12-year-old girl was admitted to our hospital with a diagnosis of major depressive disorder with psychotic features. She presented with a depressed mood, lack of interest, irritability, and repeated self-injurious behaviors or suicide attempts, including arm cutting, jumping from buildings, jumping into rivers, overdosing on medication, and attempted hanging. Moreover, she also experienced auditory hallucinations, predominantly critical in nature, with some voices issuing imperative commands, such as telling her to die. She believed that those around her intended to harm her and felt the urge to harm others in return. Her laboratory results and detailed treatment regimen are listed in Tables 1 and 2, respectively. On December 14, 2023, due to her irritable mood and aggressive behaviors, we added lithium carbonate extended-release tablet 0.3 g twice daily to her treatment regimen. However, she complained of polydipsia and polyuria during the daytime, along with enuresis every 3 to 5 days at night, as well as lumbar pain. We conducted a urinary ultrasound and spine digital radiography, but no abnormalities were detected (see Fig 1). Since the results of her urological workup were negative, we began to suspect the possibility of medication-induced enuresis. We attempted to decrease the dose of clonazepam to 1 mg at night, but did not observe significant improvement in her enuresis.

**Table 1:** Laboratory results and clinical symptom scores at different timepoints.

Test item	November 10, 2023	January 5, 2024	February 29, 2024	August 1, 2024	October 3, 2024
CBC	Normal	Normal	Normal	Normal	Normal
Liver function	Normal	Normal	Normal	Normal	Normal
Renal function	Normal	Normal	Normal	Normal	Normal
Thyroidal function	Normal	Normal	Normal	Normal	Normal
PRL	595.6 uIU/mL↑	1574.53 uIU/mL↑	74.99 uIU/mL	76.76 uIU/mL	112.76 uIU/mL
ECG	Normal	Sinus tachycardia	Normal	Normal	Normal
EEG	Normal	Normal	×	×	×
Brian MRI	Normal	×	×	×	×
Urinary ultrasound	×	Normal	×	×	×
Spine DR	×	Normal	×	×	×
Blood lithium	×	0.6 mmol/L	×	1.28 mmol/L	0.35 mmol/L
SDS	89↑	83↑	×	×	×
SAS	75↑	70↑	×	×	×
BPRS	47↑	51↑	×	×	×

× = no test.

EEG = electroencephalogram, BPRS = brief psychiatric rating scale, CBC = complete blood count, DR = digital radiography, ECG = electrocardiogram, MRI = magnetic resonance imaging, PRL = prolactin, SAS = self-rating anxiety scale, SDS = self-rating depression scale.

**Table 2:** Timeline of treatment regimens and clinical symptom changes at key time points.

	November 10, 2023	December 14, 2023	January 5, 2024	February 29, 2024	April 11, 2024	May 9, 2024	June 6, 2024	August 1, 2024	August 26, 2024	October 3, 2024	December 7, 2024
Sertraline	150 mg qd	150 mg qd	×	×	×	×	×	×	×	×	×
Risperidone	2.5 mg bid	2.5 mg bid	×	×	×	×	×	×	×	×	×
Trazodone	100 mg qn	100 mg qn	100 mg qn	50 mg qn	50 mg qn	100 mg qn	×	×	×	×	×
Clonazepam	2 mg qn	2 mg qn	×	×	×	1 mg qn	1 mg qn	1.5 mg qn	1.5 mg qn	1 mg qn	0.5 mg qn
Lithium carbonate ER	×	0.3 g bid	×	×	×	×	0.3 g bid	0.6 g bid	0.6g bid	×	×
Sodium valproate ER	×	×	500 mg bid	500 mg bid	1000 mg qn	1000 mg qn	×	×	×	×	×
Citalopram	×	×	40 mg qd	40 mg qd	40 mg qd	40 mg qd	40 mg qd	×	×	×	×
Aripiprazole	×	×	20 mg qn	20 mg qn	20 mg qn	20 mg qn	20 mg qn	20 mg qn	20 mg qn	20 mg qn	20 mg qn
Benzhexol	2 mg bid	2 mg bid	2 mg bid	2 mg bid	2 mg bid	2 mg bid	2 mg bid	2 mg bid	2 mg bid	2 mg bid	×
Mirtazapine	×	×	×	×	×	×	15 mg qn	7.5 mg qn	7.5 mg qn	×	×
Quetiapine	×	×	×	×	×	×	×	×	×	50 mg qn	100 mg qn
MECT	9 times	×	9 times	×	×	×	×	×	×	×	×
Enuresis		☆					☆	☆	☆		
Depressive mood	+++	++	+++	++	+	+	++	+	+	+	+
Auditory hallucination	√		√								
Persecutory delusion	√		√								
Self-injury or suicide	√	√	√				√				
Aggressive behavior		√					√	√	√		

☆ indicates enuresis timepoints; × indicates no use; √ indicates the presence of symptom; + indicates mild depression; ++ indicates moderate depression; +++ indicates major depression.

ER = extended release, MECT = modified electroconvulsive therapy.

On January 5, 2024, she experienced a second major depressive episode with psychotic symptoms. She predominantly presented with a severe depressed mood, irritability, and recurrent suicidal behaviors. Given her unstable clinical symptoms and a high blood prolactin level of 1574.53 uIU/mL, we made substantial changes to her treatment regimen, which included Trazodone 100 mg/d, Sodium valproate extended release 1000 mg/d, Citalopram 40 mg/d, and Aripiprazole 20 mg/d. Until May 9, 2024, she attended regular follow-up visits, during which her medication dosages were adjusted slightly based on changes in her clinical symptoms. Interestingly, she did not experience any recurrence of nocturnal enuresis during this period. On June 6, 2024, she experienced a moderate depressive episode accompanied by self-injury and aggressive behaviors. Her treatment regimen was readjusted to include clonazepam 1 mg/day, lithium carbonate extended-release tablets 0.6 g/day, citalopram 40 mg/day, aripiprazole 20 mg/day, and mirtazapine 15 mg/day. Intriguingly, her nocturnal enuresis relapsed, occurring once every 1 to 2 days. After reviewing the specifications of the medications she was taking, examining her medication history, and consulting the literature on medication-related enuresis, we suspected that her nocturnal enuresis might be linked to lithium carbonate, despite the absence of signs of neurotoxicity and a blood lithium concentration of 1.28 mmol/L. As expected, 1 week after discontinuing lithium carbonate on October 3, 2024, her nocturnal enuresis resolved, and her most recent blood lithium concentration was 0.35 mmol/L (see Tables 1 and 2).

During a 4-month follow-up period ending on December 7, 2024, she did not experience any recurrence of nocturnal enuresis. Interestingly, her clinical symptoms mostly resolved, and she expressed a desire to return to school. From a retrospective perspective, her history of Lithium carbonate use and the onset of enuresis completely overlapped (see Table 2), further confirming our hypothesis.

## 3. Discussion

Although we cannot completely rule out the combined effect of lithium carbonate extended-release tablets with other psychotropics on nocturnal enuresis in our case, as these medications have been reported to be associated with enuresis, our thorough review of the case revealed a strong association between the patient’s nocturnal enuresis and the use of lithium carbonate extended-release tablets. Her nocturnal enuresis completely overlapped with the medication use and disappeared after it was discontinued. Lithium carbonate extended-release tablets have several advantages compared to conventional lithium carbonate, including more stable blood drug concentrations, fewer adverse events, and less frequent dosing.^[18]^ To the best of our knowledge, no reports have documented nocturnal enuresis associated with lithium carbonate extended-release tablets.

The most common urological side effects of lithium include polyuria and polydipsia.^[15]^ Previous studies have reported that lithium can cause nephrogenic diabetes insipidus, which may be linked to reduced kidney sensitivity to vasopressin^[19]^ and impaired expression of aquaporin-2 in the collecting duct principal cells,^[20]^ leading to impaired water reabsorption. A case report suggested that lithium may enhance renal prostaglandin activity, leading to polyuria.^[21]^ In our case, the patient exhibited significant polyuria and polydipsia during the day and enuresis at night. We postulate that her nocturnal enuresis might be a continuation of daytime polyuria, influenced by the sedative effects of other psychotropic medications.

Since various types of psychotropic medications, including antipsychotics, antidepressants, anticonvulsants, and benzodiazepines, have been reported to be associated with enuresis under specific conditions, medication-induced enuresis should be considered, particularly when these medications are used in combination. Furthermore, individual differences in response to psychotropics should be taken into account, especially when a rare side effect emerges. Interestingly, there are reports that antidepressants, such as imipramine^[22]^ and fluvoxamine,^[23]^ can be used to treat enuresis. Undoubtedly, these contradictory effects make this condition more complicated. We suspect that these opposing effects may be related to different pathophysiological mechanisms.

## 4. Conclusion

Although lithium carbonate extended-release tablets are relatively safe with strict monitoring of blood lithium concentrations, individual differences in response to lithium may contribute to nocturnal enuresis, particularly when combined with other psychotropics. It is important to be aware of the possibility of lithium-induced nocturnal enuresis when polyuria and polydipsia emerge. Furthermore, the risk of nocturnal enuresis may increase when lithium is combined with other psychotropics.

## Acknowledgments

The authors would like to thank the patient for her cooperation.

## Author contributions

**Conceptualization:** Bo Liu.

**Project administration:** Youguo Tan.

**Resources:** Yanping Feng, Qingqing Xiang.

**Writing – original draft:** Bo Liu.

**Writing – review & editing:** Yanping Feng, Qingqing Xiang, Xianzhen Dai, Pingping Ma, Youguo Tan.
